# SASE, Success and Adverse event Score in Endoscopic Retrograde Cholangiopancreatography: a Novel Grading System

**DOI:** 10.1186/s12876-023-02942-w

**Published:** 2023-09-15

**Authors:** Andreas Maieron, Christine Duller, Andreas Püspök, Emanuel Steiner, Christine Kapral

**Affiliations:** 1 Karl Landsteiner University of Health Sciences, Dr. Karl-Dorrek-Straße 30, Krems, 3500 Austria; 2 Division of Internal Medicine, Gastroenterology & Hepatology, University Hospital of St. Pölten, Dunant-Platz 1, St. Pölten, A - 3100 Austria; 3 Quality Assurance Working Group: Benchmarking ERCP, Austrian society of Gastroenterology and Hepatology, Freyung 6, Vienna, 1010 A Austria; 4 Institute of Applied Statistics, Johannes Kepler University Linz, Altenberger Strasse 69, Linz, A - 4040 Austria; 5 Department of Internal Medicine 2, St. John´s Hospital Eisenstadt, Johannes von Gott-Platz 1, Eisenstadt, A - 7000 Austria; 6 Department of Gastroenterology and Hepatology, Ordensklinikum Barmherzige Schwestern, Seilerstätte 4, Linz, A - 4020 Austria

**Keywords:** ERCP, Benchmarking, Quality assessment score, Adverse events, Treatment success

## Abstract

**Background:**

Validated, accepted grading tools for preprocedural complexity assessment in ERCP are lacking. We therefore created a grading system for ERCP based on the classification used by the American Society for Gastrointestinal Endoscopy (ASGE).

**Methods:**

Data on ERCP adverse events (AE) and success were collected in a multicenter, prospective uncontrolled study. Multiple logistic regressions were applied to success and AEs in accordance with the ASGE classification. Each procedure suggested by ASGE was tested against different outcomes. Results were used to create a score and were evaluated in a control cohort.

**Results:**

16,327 ERCPs were documented in 27 centers. Analysis of ASGE categorization (10,904 cases) showed that this model fails to adequately predict parameters of complexity; only for cardiopulmonary AEs and perforation was no significant variance evident. Depending on the specific clinical circumstances, probability of success of the intervention sometimes varied significantly in risk, implying a twofold score, one part for probability of success and one for risk. A split score with three levels each was designed and tested in a validation cohort (5,423 procedures). Achieving therapeutic targets / post-ERCP pancreatitis could be correctly predicted in 87.0%/95.3%.

**Conclusions:**

Grading ERCP success and AEs have to be considered independently. Onefold grading systems appear incomplete and unable to provide an adequate classification of severity. SASE (Success and Adverse Event Score in Endoscopic Retrograde Cholangiopancreatography) was created to incorporate these findings. Showing high predictive value, this score could be a potent tool for planning ERCP and training in endoscopy.

**Supplementary Information:**

The online version contains supplementary material available at 10.1186/s12876-023-02942-w.

## Background

Endoscopic retrograde cholangiopancreatography (ERCP) has evolved from a diagnostic tool to a highly complex intervention [1]. This challenging examination can be associated with potential life-threatening adverse events (AE) connected to the procedure itself and the therapeutic measures carried out [2, 3]. Due to the relatively high risk of procedure-related AEs, quality assessment of ERCP has become a main focus of endoscopists in recent years [4]. Several working groups have proposed quality indicators for ERCP to improve the procedure’s success and minimize side-effects. Such benchmarks would allow the graduation and comparability of the interventions and therefore the implementation of quality assurance programs [5–9]. Adequate training and experience of the endoscopist certainly play an important role in the patient’s outcome [10]. However, little is currently known about the impact of technical difficulty on both the success and the AEs associated with ERCP.

Schutz and Abbott [11] were the first to address the complexity issue of ERCP, suggesting a five-point scale to assess the “level of difficulty”. In 2011 a working party of the American Society for Gastrointestinal Endoscopy (ASGE) published a grading system for the complexity of all endoscopic procedures including ERCP [12]. Their goal was to focus on circumstances in which difficulty is predictable such as known altered anatomy. The ASGE working party provided a list of specific endoscopic techniques, clinical contexts and some anatomical / pathological challenges. This list was sent out to expert endoscopists for scoring on a four-point scale and assessing additional aggravating factors (age < 3, outside normal working hours, etc.). After 76 replies were returned from 230 contacted specialists and the list of procedures and contexts was segregated into four levels based on the median score provided by the experts. This scale should provide information on the difficulty of the planned procedure and therefore the technical success rate. To date the ASGE classification has had a meaningful impact on benchmarking in ERCP throughout the world [5, 13]. Currently a US working group is creating a training supervision tool for endoscopic ultrasound and ERCP, The EUS and ERCP Skills Assessment Tool (TEESAT), where complexity assessment of the procedures is based on the ASGE classification system [13]. In this context ASGE recommends TEESAT and therefore the classification system of Cotton et al. for further use and additional evaluation to improve quality in endoscopy [13].

In 2006, the Austrian Society of Gastroenterology and Hepatology (ÖGGH) initiated a nationwide voluntary benchmarking program for ERCP [10]. In 2010, 118 institutions performed an estimated 13,000–15,000 ERCP procedures [5]. Approximately 2,700 ERCP / year were documented (a fifth of all performed procedures in Austria / year) and give an overview of both academic and community-based services with varying caseloads and expertise.

A comprehensive evaluation of the ASGE grading system, as recommended by the working party itself, has yet to take place. The use of this classification system in TEESAT, a broadly recommended and used tool, seems to make an examination of this score all the more interesting. Our study therefore aimed to assess the recommended grading system on the basis of the data collected by the ÖGGH regarding success and AE rate and to adjust the metrics to create a novel scoring system.

We therefore gathered data from the Austrian national survey for quality assessment of ERCP over a four-year period from 2013 to 2016 (10,904 cases). Moreover, we introduced a validation cohort (5,423 cases) from 2017 to 2018 to test our new model.

## Methods

This study represents a prospective uncontrolled large-scale multicenter data collection. In 2013, the ÖGGH executive committee for benchmarking in ERCP updated their online questionnaire to assess the complexity of ERCP according to the ASGE recommendations of 2011 (corresponding frequencies are shown in Additional file 1). Recognized quality indicators as provided by the European and the American Society of Gastroenterology were collected accordingly [3, 6, 14, 15]. AEs were also reported: all patients were followed up for at least 48 h and laboratory parameters were collected to confirm /exclude cholangitis, post-ERCP pancreatitis (PEP), perforation, cardiopulmonary AEs and bleeding [16, 17]. All centers participating in the survey were asked to report on every ERCP performed to obtain the best-quality data. However, both the participation of each center and the recording of each ERCP were voluntary. The Institute of Applied Statistics, Johannes Kepler University Austria (IFAS-JKU), conducted the web-based questionnaire for data recording. The IFAS-JKU provided unique access codes for entering each individual procedure into the data mask. Both the participating endoscopist and the patients remained anonymous. After confirmation, all submitted datasets were transferred to the IFAS-JKU for statistical analysis.

### Outcome and definitions

The primary outcome was an evaluation and reorganization of the list of specific endoscopic interventions [12], taking into account their complexity. For this the success and AE rate of all ERCPs were recorded by the IFAS- JKU and graded accordingly to the cotton system [12].

#### Success

The success of the procedure was defined as visualization and cannulation of the desired duct and achievement of the therapeutic target [6, 18]. The success rate therapeutic target achievement was classified as entirely (yes), partially and not (both counted as “no” in analysis) achieved [18], the success rates for visualization and cannulation were binary (yes/no).

#### Adverse events

PEP was characterized according to the definition initially developed by Cotton et al. in 1991 [19] and the modifications by Freeman et al. [2], as follows: Abdominal pain developing or worsening, accompanied by at least a threefold increase in pancreatic enzymes 24 to 48 h after the intervention. Post-ERCP cholangitis was defined as follows: Increased body temperature > 38.0° C; leucocytosis and abdominal pain. Radiologically documented retro- or free intraperitoneal air after ERCP defined a perforation. Moreover, the total bleeding rate was evaluated. A bleeding requiring blood transfusion and/or intervention and/or Hb-reduction of at least 3 g/dl was defined as clinically relevant [2, 4, 19]. Cardiopulmonary AEs were defined as a drop in systolic blood pressure during the examination of < 90 mmHg for more than five minutes, oxygen desaturation of < 90% for more than five minutes, unplanned intubation or resuscitation [16].

Difficult cannulation was defined as failure of deep cannulation of the desired duct after 10–15 attempts or taking more than 10 min to complete [14].

### Statistical analysis

In a first step, we ran bivariate analyses for relevant parameters of outcome (success and AEs) with the levels of difficulty according to the ASGE grading system, including the specific endoscopic indications / procedures within those levels. To gain a deeper insight into which items of specific endoscopic techniques, clinical contexts and challenges have an influence on success (cannulation of the desired duct and achievement of the therapeutic target) or AEs (AEs with an occurrence of > 1% in our cohort: at least one AE, bleeding and PEP) we then calculated a logistic regression model for each item of success and AE.

Cases showing missing information were not completely excluded except for the corresponding analysis. Therefore, the number of valid cases can differ slightly for different analyses.

We evaluated our models in a validation cohort; each regression model was used to predict the specific outcome for the additional ERCPs recorded in 2017 and 2018.

Based on these results a score with three levels for success and AE was generated, called Success and Adverse Event Score in Endoscopic Retrograde Cholangiopancreatography (SASE).

In a final step, Spearman correlations between parameters of success (achievement therapeutic target/cannulation) and success score, as well as between parameters of AEs (bleeding, PEP, at least one complication) and adverse event score were calculated.For statistical analyses, IBM SPSS Statistics for Windows, Version 25.0 (IBM, Armonk, New York) and R: A Language and Environment for Statistical Computing (The R Foundation for Statistical Computing, Vienna) were used, using the usual level of significance (5%).

### Compliance with ethical standards

All procedures performed in this study involving human participants complied with the ethical standards of the institutional research committee and the 1964 Declaration of Helsinki declaration and its later amendments or comparable ethical standards. All samples were properly masked for the performance of this trial. This study was approved by the Review Board of the Austrian Society of Gastroenterology and Hepatology.

## Results

Between 2013 and 2016, 26 institutions participated in the evaluation. A total of 10,904 procedures (40–1,390 per center) were reported, performed by endoscopist from different disciplines including trainees. The vast majority (89.9%) were therapeutic procedures, followed by therapeutic and diagnostic (5.0%) and solely diagnostic (3.0%).

Baseline characteristics are presented in Table 1. The patients’ ages ranged from 0 to 103 years, including two children younger than three years. For statistical reasons these two cases (rare events, 0.02% of all cases) had to be omitted from the regression analyses. Regarding comorbidities, women showed significantly more cardiac insufficiency than men, while men showed more coronary heart disease and cases of liver cirrhosis. Respiratory failure was not significantly different between genders (overall comorbidities are presented in Table 1). Distribution of AEs according to the ASGE grading system is shown in Additional file 2.

**Table 1:** Baseline characteristics of model and validation cohorts; correct prediction of SASE (percentage) in the validation cohort

	Model cohort (n = 10,904)	Validation cohort (n = 5423)	Correctly predicted by SASE (%)
Basic data female/male			
Percent (%)	52.3/47.7	49.0/51.0	
Mean age (± standard deviation)	70.2 (± 17.4)/68.3 (± 14.9)	70.6 (± 17.1)/69.0 (± 14.6)	
Comorbidities overall (%)			
Cardiac insufficiency	11.6	12.1	
Coronary heart disease	10.8	12.5	
Respiratory failure	3.7	2.8	
Cirrhosis	2.1	1.8	
Success overall (%)			
Cannulation of desired duct	92.5	93.3	93.2
Achievement of therapeutic target	86.9	86.8	87.0
AE overall (%)			
Post ERCP pancreatitis	3.5	4.7	95.3
Bleeding	3.7	4.8	95.2
At least one AE	9.5	11.2	88.8

SASE, Success and Adverse Event Score in Endoscopic Retrograde Cholangiopancreatography.

Data in mean and standard deviation.

At least one adverse event occurred in 9.5% of valid cases; 6.8% for grade 1, 10.9% for grade 2; 8.4% for grade 3; and 11.5% for grade 4 (p = 0.000). The overall PEP, bleeding, and cholangitis rate was as follows 3.5%; 3.7%; 1.0%. Cardiopulmonary AE was recorded in 0.8%, a perforation in 0.6% and other AEs in 1.0% of valid cases (details are shown in Additional file 2).

Success rates for each procedure in each category according to the ASGE grading system and the overall AE rate for each procedure were assessed (details are shown in Additional file 3 and Table 2 respectively).

**Table 2:** AE rates in our cohort subdivided according to the ASGE grading system

		Bleeding	PEP	At least one adverse event
**ERCP**	**Cases**	**Valid%**	**Valid%**	**Valid%**
Billroth II	179	2.2%	4.0%	8.5%
Emergency case (outside normal hours)	525	4.6%	2.5%	10.1%
Child < 3 years	2	0.0%	50.0%	50.0%
Previous failed/incomplete procedure	304	5.6%	2.6%	12.0%
Level 1				
Deep cannulation of duct of interest, main papilla sampling	1328	3.9%	3.3%	8.4%
Aspiration of bile	148	2.7%	3.4%	7.4%
Biliary stent removal /exchange	760	1.2%	1.1%	4.6%
Level 2				
Biliary stone extraction < 10 mm	3627	5.0%	3.4%	10.8%
Treat biliary leaks	208	2.4%	2.4%	7.7%
Treat extrahepatic benign / malignant strictures	1849	3.9%	4.6%	11.1%
Place prophylactic pancreatic stents	232	7.3%	10.3%	18.6%
Level 3				
Biliary stone extraction > 10 mm	1408	4.1%	2.3%	9.1%
Minor papilla cannulation in p. divisum, and therapy	79	1.3%	12.7%	15.2%
Remove of internally migrated biliary stents	73	0.0%	1.4%	9.6%
Intraductal imaging, biopsy	156	1.3%	3.2%	7.7%
Manage acute or recurrent pancreatitis	428	2.1%	5.1%	8.2%
Treat pancreatic strictures	345	0.6%	6.4%	7.9%
Remove pancreatic stones mobile and < 5 mm	129	0.0%	7.0%	7.8%
Treat hilar tumors	193	1.6%	2.1%	5.3%
Treat benign biliary strictures hilum and above	390	0.8%	2.3%	5.7%
Manage suspected sphincter Oddi dysfunction	24	4.2%	41.7%	41.7%
Level 4				
Extract internally migrated pancreatic stent	32	0.0%	3.1%	6.3%
Intraductal image guided therapy (e.g. PDT; electrohydraulic lithotripsy)	93	1.1%	2.2%	10.8%
Pancreatic stones impacted and/or > 5 mm	58	3.4%	10.3%	15.5%
Intrahepatic stones	76	2.6%	2.6%	6.6%
Ampullectomy	51	15.7%	11.8%	28.0%
ERCP after Whipple or Roux - en - Y	125	0.0%	0.8%	8.2%
All Cases (**NOT** sum - multiple answers possible)	10,917	3.7%	3.5%	9.5%

Minima and Maxima highlighted; Abbreviations: ERCP, endoscopic retrograde cholangiopancreatography; PDT, photodynamic therapy; PEP, post ERCP pancreatitis.

Achievement of the therapeutic target ranged from 46.4% in pancreatic stones impacted and/or > 5 mm to 96.8% in extracting an internally migrated pancreatic stent. The success rate for cannulation of the desired duct ranged from 54.6% in ERCP post Whipple procedure to 100.0% in Sphincter of Oddi dysfunction (SOD) patients.

The overall AE rate (at least one AE) varied from 4.6% in patients with biliary stent removal /exchange to 41.7%. The highest AE rates were observed in patients with SOD (41.7%), papillectomies (28.0%), prophylactic pancreatic stenting (18.6%), pancreatic stones > 5 mm (15.5%) and minor papilla cannulation (15.2%). It is notable that the AE rate in SOD patients is caused by PEP in all cases, in contrast to papillectomies (overall AE rate: 28.0%, PEP: 11.8%, bleeding 15.7%), where a significant proportion of AEs was clearly a result of bleeding.

Cannulation of the desired duct and the achievement of the therapeutic target could not be predicted correctly by the ASGE classification (p < 0.01). This was also true of the following AEs: PEP (p < 0.05), post-ERCP cholangitis (p < 0.05) and bleeding (p < 0.01). Cardiopulmonary AEs and perforation displayed no significant discrepancy. The probability of having at least one of the mentioned AEs also differed significantly from the estimate of the scoring system (p < 0.01).

**Fig. 1:**
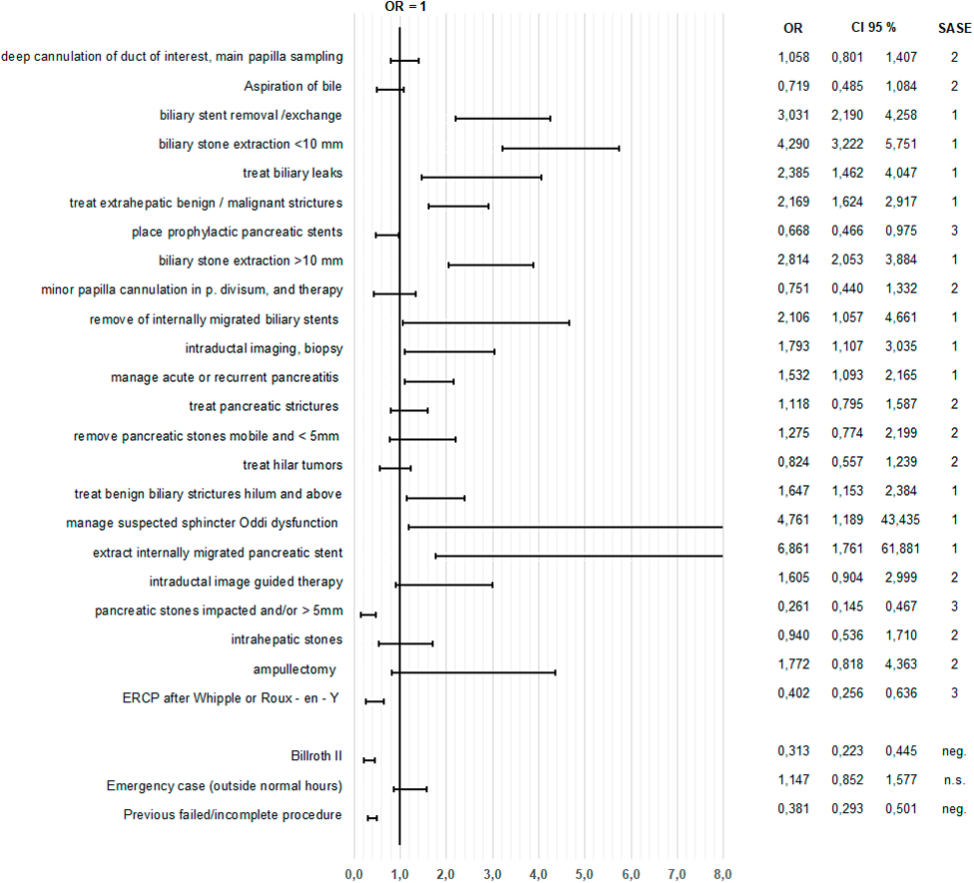
Achievement of therapeutic target: Odds Ratios (OR), 95% Confidence Intervals (CI), Success and Adverse Event Score in Endoscopic Retrograde Cholangiopancreatography (SASE). CI truncated at 8 (for better visibility), CI left of 1 shows significant results (negative / SASE 3 = low success), CI covering 1 shows non-significant results (n.s. / SASE 2), CI right of 1 shows significant results (positive / SASE 1 = high success)

Table 3 presents the odds ratio (OR) and 95% confidence interval (CI) for parameters of success and AEs for each intervention mentioned in the ASGE classification: an OR and CI > 1 are defined as 1 (low risk of failure / AE), an OR and CI < 1 as 3 (high risk of failure / AE) and parameters with an CI overlapping the OR of 1 are defined as 2 (intermediate risk of failure / AE), shown as an example in Fig. 1.

**Table 3:** Success and Adverse Event Score in Endoscopic Retrograde Cholangiopancreatography (SASE), regrading of the ASGE complexity grades for ERCP

	Success			Adverse event				
	Target	Cannu-lation	Overall^a^ success	Bleeding	PEP	At least one	Overall^a^ compli-cation	ASGE grading system
Billroth II	neg.	neg.		n.s.	n.s.	n.s.		neg.
Emergency case (outside normal hours)	n.s.	n.s.		n.s.	n.s.	n.s.		neg.
Child < 3 years	n.a.	n.a.		n.a.	n.a.	n.a.		neg.
Previous failed/incomplete procedure	neg.	neg.		pos.	n.s.	n.s.		neg.
SASE 1/1								
Biliary stent removal /exchange	1	1	**1**	1	1	1	**1**	1
SASE 1/2								
Treat benign biliary strictures hilum and above	1	1	**1**	1	2	2	**2**	3
Biliary stone extraction > 10 mm	1	1	**1**	2	2	2	**2**	3
Extract internally migrated pancreatic stent	1	1	**1**	2	2	2	**2**	4
Intraductal imaging, biopsy	1	1	**1**	2	2	2	**2**	3
Manage acute or recurrent pancreatitis	1	1	**1**	2	2	2	**2**	3
Remove of internally migrated biliary stents	1	1	**1**	2	2	2	**2**	3
Treat biliary leaks	1	1	**1**	2	2	2	**2**	2
SASE 1/3								
Biliary stone extraction < 10 mm	1	1	**1**	3	2	3	**3**	2
Manage suspected sphincter Oddi dysfunction	1	1	**1**	2	3	3	**3**	3
Treat extrahepatic benign/malignant strictures	1	1	**1**	2	3	3	**3**	2
SASE 2 /2								
Deep cannulation of duct of interest, main papilla sampling	2	1	**2**	2	2	2	**2**	1
Aspiration of bile	2	2	**2**	2	2	2	**2**	1
Intraductal image guided therapy (e.g. PDT; electrohydraulic lithotripsy)	2	1	**2**	2	2	2	**2**	4
Intrahepatic stones	2	1	**2**	2	2	2	**2**	4
Minor papilla cannulation in p. divisum, & therapy	2	2	**2**	2	3	2	**2**	3
Remove pancreatic stones mobile and < 5 mm	2	1	**2**	2	2	2	**2**	3
Treat hilar tumors	2	1	**2**	2	2	2	**2**	3
Treat pancreatic strictures	2	1	**2**	1	3	2	**2**	3
SASE 2/3								
Ampullectomy	2	1	**2**	3	3	3	**3**	4
Pancreatic stones impacted and/or > 5 mm	3	1	**2**	2	3	3	**3**	4
SASE 3/2								
ERCP after Whipple or Roux - en - Y	3	2	**3**	2	2	2	**2**	4
SASE 3/3								
Place prophylactic pancreatic stents	3	2	**3**	3	3	3	**3**	2

neg. significant negative OR, corresponding to lower success/adverse event rate

pos. significant positive OR, corresponding to higher success/ adverse event rate

SASE grade 1: high success/low adverse event rate

SASE grade 2: intermediate success/ adverse event rate

SASE grade 3: low success/high adverse event rate

Abbreviations: ERCP, endoscopic retrograde cholangiopancreatography; n.a., not available; n.s., not significant; PDT, photodynamic therapy; PEP, post ERCP pancreatitis.

^a^median

The multivariate analysis of our data showed irregularities between success and occurrence of AEs (Table 3). This made it necessary to differentiate between these two factors for a more reliable classification and resulted in a twofold score with three levels for both, called Success and Adverse Event Score in Endoscopic Retrograde Cholangiopancreatography (SASE).

According to this new scoring system, the probability for success is high for grade 1, intermediate for grade 2, and low for grade 3. For AEs, grade 1 means low risk, grade 2 intermediate risk, and grade 3 high risk. Regarding success, only prophylactic pancreatic stenting and ERCP after a Whipple procedure falls into level 3. Of AEs, biliary stone extraction < 10 mm, extrahepatic stenosis, prophylactic pancreatic stenting, management of suspected sphincter of Oddi dysfunction, pancreatic stone extraction (> 5 mm) and papillectomy fall into level 3. This means the placement of prophylactic pancreatic stents has a low probability of success and a high risk of AEs. It is therefore rated SASE 3/3. The full scoring system is shown in Table 3.

Of the additional complicating circumstances (intervention outside normal working hours, altered anatomy, failed attempt in the past) altered anatomy, e.g. Whipple, and failed attempt in the past showed a significant negative impact on success.

### Validation cohort

In 2017 and 2018, 5,423 ERCPs were registered in 27 centers and established as a control cohort. Baseline characteristics, comorbidities and the percentage of complexity parameters correctly predicted by our model are shown in Table 1.

Achievement of the therapeutic target was predicted correctly in 87.0% of cases in modeling data (2013–2016) and in 87.0% of cases in evaluating data (2017–2018). For visualization and cannulation, results were correctly predicted for 92.6% of cases in modeling data and 93.2% in evaluating data. Regarding AE rates, PEP showed a correct prediction rate of 96.5% in modeling data and 95.3% in evaluating data.

Correct prediction of bleeding was possible in 96.3% of cases in modeling data and 95.2% in evaluating data, and for at least one AE the rate was 90.5% in modeling data and 88.8% in evaluating data.

### Spearman correlation

Significant correlations between both parameters of success (achievement therapeutic target/cannulation) and success score, as well as between all parameters of AEs (bleeding, PEP, at least one complication) and adverse event score were found in our model and validation cohort, respectively (PEP validation cohort p = 0.04, all others p < 0.001). Presented in Additional file 4 in detail.

## Discussion

The ASGE classification system by Cotton et al. [12] is used to grade complexity [10, 13], manage ERCP resources and monitor the progress of trainees in endoscopy.

In this study, we are able to give a deep insight into both success and AE in an unselected cross-sectional cohort of over 10,904 ERCPs performed in 26 different institutions ranging from primary to large tertiary academic centers in Austria. Moreover, we were able to confirm our model with a validation set of 5,423 ERCPs. The findings of our study indicate that the grading system suggested by the ASGE does not adequately predict the outcome of ERCP (success / AE). Of the tested parameters only cardiopulmonary AEs and perforation showed no discrepancy, while all other parameters of success and AEs significantly differed from the ASGE grading. Therefore, the classification was regraded using the OR calculated for the major aspects of success and AE in respect of the specific clinical situations.

We were able to show some striking differences: The extraction of biliary stones > 10 mm seems technically difficult; however, the therapeutic target can be achieved in most cases and the AE rate classed as medium. This may also be attributed to emerging techniques, their improvement and increasing popularity such as cholangioscopy and balloon dilatation. Novel techniques like transpancreatic sphicterotomy or needle-knife techniques may also contribute to the numbers mentioned above [20, 21].

The overall AE rate in this study was in line with other large – scale analyses [6, 22] and review articles such as the work of Andriulli et al. 2007. In this analysis of over 16,000 patients the overall AE rate was approximately 7%, and PEP occurred in 3.5% of all cases [23]. Compared to our results, however, it is notable that some procedures classified as easy in different grading systems [11, 12, 24, 25] had unexpectedly high AE rates. Conversely, some of those categorized as complex showed high success accompanied by low AE rates. As an example, the prophylactic stenting of the pancreas was classified as level 2 by Cotton et al., but showed the lowest degree of success and a high AE rate in our analysis. Certainly, the high AE rate in prophylactic pancreatic stenting might be due to the difficulty of placing the stent or the complexity of the underlying procedure itself. This might be one of the reasons why the ESGE recommends administering NSAIDs to all patients without contraindication for the first – line prevention of PEP. Pancreatic stenting should only be performed when necessary and, if it can be accomplished easily [26]. On the other hand, the extraction of internally migrated pancreatic stents was assigned to level 4, but showed a high success and only an average AE rate [12].

A complexity grading scale by Schultz et al. [11, 22] showed a correlation with success, but no differences regarding the AE rate in a relatively small dataset of approximately 200 patients. Moreover, this grading system includes diagnostic ERCP, which has been largely abandoned in favor of magnetic resonance cholangiopancreatography, and consequently now seems somewhat outdated.

Ragunath et al. [25] developed a grading system mainly for educational purposes that included 305 procedures. A correlation of success in trainees, but not in trainers could be shown. More importantly the system was unable to prove significant differences in the AE rates throughout the different complexity grades.

In 2017, Olsson et al. [24] introduced the H.O.S.E. classification, based on approximately 2,000 ERCPs. The classification consists of three different classes. A clear correlation between grading and time of the procedure could be shown, and to some extent there was even a correlation with AEs. However, the score might be biased by the fact that the data is monocentric, coming from the Karolinska University Hospital which is a tertiary referral center and a large teaching institute for ERCP. This might contribute to higher AE rates [27]. Furthermore, the H.O.S.E. score contains peri-procedural criteria and therefore cannot be used as a tool to direct ERCPs towards referral centers.

In a smaller Turkish trial [28] encompassing approximately 1,000 ERCPs the ASGE grading system was analyzed showing a correlation of success and AEs throughout the different grades. However, the design of the study was monocentric and the number of endoscopists limited, which means that the results might not be transferable to a large population.

A validated grading system for success and AEs seems to be important to assess a patient’s individual risk before the intervention. An appropriate grading system could be valuable for managing resources and determining the equipment and expertise required by endoscopists.

Furthermore, training could be planned on both probability of success and AEs. By choosing the right level of complexity according to the trainees’ skills, the success of the trainee could be maximized by diminishing the patient´s risks. Such a score could further improve training evaluation and help with establishing standardized training programs [13]. Based on the preprocedural assignment of the ERCP, complex procedures or those with a high risk of AEs could be referred directly to a tertiary center. An evaluated grading system could serve as a reference in benchmarking programs at different endoscopic centers. Institutional and individual success and AE rates could easily be compared for each level.

The strength of this study is the large number of ERCPs performed in primary, secondary and tertiary sites with different caseloads, giving us a good insight into everyday clinical practice in Austria. A selection bias based on referral is unlikely [27]. Still, this study presents an uncontrolled prospective survey with several limitations. It should be noted that the survey is based on voluntary participation, with uncertain data completeness. This might be especially true for late AEs. To determine the accuracy of the reported data, over 1,000 ERCPs were clinically reviewed during data collection. In a previous analysis a registration rate of 83.3% of all procedures performed at the participating centers could be shown [10]. Evaluation of expertise was not possible due to the anonymous design of the data collection. This could potentially lead to an overestimation of the ease of difficult procedures, which are mainly performed by experienced interventionists. However, we are confident of this new classification system, as we were able to demonstrate the accuracy of our models in the validation cohort. What is perhaps most important is that for AEs, e.g. PEP, correct prediction could be shown in 96.5% of cases in modeling data and 95.3% in evaluating data. To underline the strength of our classification system we also performed a Spearman correlation proving significant correlations between success/adverse event and SASE.In this study we reorganized the ASGE grading system, creating a new score that takes success and AEs into account independently. Due to the potential harm of ERCP to our patients it seems crucial to introduce an AE grading along with the success rate. The SASE includes this result consisting of three levels of difficulty and risk, aiming for a score applicable in clinical practice for colleagues of different levels of expertise.

The score should be evaluated in different cohorts including ours and other large multicenter ones, taking a close look at the case mix, AEs, and success throughout the different procedures to determine its actual significance.

### Electronic supplementary material

Below is the link to the electronic supplementary material.

Supplementary Material 1

Supplementary Material 2

Supplementary Material 3

Supplementary Material 4

## Data Availability

The datasets used and/or analysed during the current study are available from the corresponding author on reasonable request.

## Abbreviations

AE: Adverse events
ASGE: American Society for Gastrointestinal Endoscopy
CI: 95% confidence interval
EHL: Electrohydraulic lithotripsy
ERCP: Endoscopic retrograde cholangiopancreatography
IFAS-JKU: Institute of Applied Statistics, Johannes Kepler University Austria
NSAID: Non-steroidal anti-inflammatory drug
ÖGGH: Austrian society of Gastroenterology and Hepatology
OR: Odds ratio
PDT: Photodynamic therapy
PEP: Post ERCP pancreatitis
SASE: Success and Adverse Event Score in ERCP
SOD: Sphincter of oddi dysfunction
TEESAT: The EUS and ERCP Skills Assessment Tool
