# Supplementary material for: SASE, Success and Adverse event Score in Endoscopic Retrograde Cholangiopancreatography: a Novel Grading System
Source: BMC Gastroenterol. 2023 Sep 15;23:314. doi: 10.1186/s12876-023-02942-w (PMC10504789; doi:10.1186/s12876-023-02942-w)
Supplement: Supplementary file 1 — Supplementary Material 1 [file 12876_2023_2942_MOESM1_ESM.docx]

**SASE, Success and Adverse Event Score in Endoscopic Retrograde Cholangiopancreatography. A Novel Grading System.**

*Andreas Maieron, MD, PhD, Christine Duller, ScD, PhD, Andreas Püspök, MD, PhD, Emanuel Steiner, MD, Christine Kapral, MD, PhD*

Corresponding author

Priv.-Doz. Dr. Andreas Maieron

Department of Internal Medicine 2; Gastroenterology & Hepatology

Karl Landsteiner University of Health Sciences, University Hospital of St. Pölten

Mühlweg, A - 3100 St. Pölten, Austria

E-Mail: Andreas.Maieron@stpoelten.lknoe.at

Additional file 1: Cases of our cohort graduated according to the ASGE grading system

| **ERCP** | **#Cases** | **Percent**^1^ |
| --- | --- | --- |
| Billroth II | 179 | 1.6 |
| Emergency case (outside normal hours) | 525 | 4.8 |
| Age < 3 years | 2 | 0.0 |
| Previous failed/incomplete procedure | 304 | 2.8 |
| Level 1 | 1913 | 17.6 |
| Deep cannulation of duct of interest, main papilla sampling | 1328 | 12.2 |
| Aspiration of bile | 148 | 1.4 |
| Biliary stent removal /exchange | 760 | 7.0 |
| Level 2 | 5213 | 47.8 |
| Biliary stone extraction <10 mm | 3627 | 33.3 |
| Treat biliary leaks | 208 | 1.9 |
| Treat extrahepatic benign / malignant strictures | 1849 | 17.0 |
| Place prophylactic pancreatic stents | 232 | 2.1 |
| Level 3 | 3074 | 28.2 |
| Biliary stone extraction >10 mm | 1408 | 12.9 |
| Minor papilla cannulation in p. divisum, and therapy | 79 | 0.7 |
| Remove of internally migrated biliary stents | 73 | 0.7 |
| Intraductal imaging, biopsy | 156 | 1.4 |
| Manage acute or recurrent pancreatitis | 428 | 3.9 |
| Treat pancreatic strictures | 345 | 3.2 |
| Remove pancreatic stones mobile and < 5mm | 129 | 1.2 |
| Treat hilar tumours | 193 | 1.8 |
| Treat benign biliary strictures hilum and above | 390 | 3.6 |
| Manage suspected sphincter Oddi dysfunction | 24 | 0.2 |
| Level 4 | 704 | 6.5 |
| Extract internally migrated pancreatic stent | 32 | 0.3 |
| Intraductal image guided therapy (eg. PDT; electrohydraulic lithotripsy) | 93 | 0.9 |
| Pancreatic stones impacted and/or > 5mm | 58 | 0.5 |
| Intrahepatic stones | 76 | 0.7 |
| Ampullectomy | 51 | 0.5 |
| ERCP after Whipple or Roux - en - Y | 125 | 1.1 |
| Based on 10904 cases; Abbreviations: ERCP, endoscopic retrograde cholangiopancreatography; PDT, photodynamic therapy | | |
