# Supplementary material for: SASE, Success and Adverse event Score in Endoscopic Retrograde Cholangiopancreatography: a Novel Grading System
Source: BMC Gastroenterol. 2023 Sep 15;23:314. doi: 10.1186/s12876-023-02942-w (PMC10504789; doi:10.1186/s12876-023-02942-w)
Supplement: Supplementary file 2 — Supplementary Material 2 [file 12876_2023_2942_MOESM2_ESM.docx]

**SASE, Success and Adverse Event Score in Endoscopic Retrograde Cholangiopancreatography. A Novel Grading System.**

*Andreas Maieron, MD, PhD, Christine Duller, ScD, PhD, Andreas Püspök, MD, PhD, Emanuel Steiner, MD, Christine Kapral, MD, PhD*

Corresponding author

Priv.-Doz. Dr. Andreas Maieron

Department of Internal Medicine 2; Gastroenterology & Hepatology

Karl Landsteiner University of Health Sciences, University Hospital of St. Pölten

Mühlweg, A - 3100 St. Pölten, Austria

E-Mail: Andreas.Maieron@stpoelten.lknoe.at

Additional file 2: Success and adverse event rates in our cohort according to the ASGE grading system

|  |  | **ASGE grading system** | | | | |  | |  | |  | |
| --- | --- | --- | --- | --- | --- | --- | --- | --- | --- | --- | --- | --- |
|  |  | 1 | 2 | 3 | | 4 | | Missing | | Valid | | Sum |
|  | #Cases | 1913 | 5213 | 3074 | | 704 | |  | | 10904 | | 10904 |
|  | Percent | 17.5% | 47.8% | 28.2% | | 6.5% | |  | | 100.0% | | 100.0% |
| Success visualization | #Valid Cases | 1845 | 5136 | 3024 | | 690 | | 209 | | 10695 | | 10904 |
|  | #Cases Success | 1606 | 4862 | 2883 | | 599 | |  | | 9950 | | 9950 |
|  | Percent within grade | 87.0% | 94.7% | 95.3% | | 86.8% | |  | | 93.0% | |  |
| Success cannulation | #Valid Cases | 1829 | 5103 | 3004 | | 686 | | 282 | | 10622 | | 10904 |
|  | #Cases Success | 1575 | 4802 | 2859 | | 592 | |  | | 9828 | | 9828 |
|  | Percent within grade | 86.1% | 94.1% | 95.2% | | 86.3% | |  | | 92.5% | |  |
| Success therapeutic target | #Valid Cases | 1865 | 5083 | 2989 | | 690 | | 277 | | 10627 | | 10904 |
|  | #Cases Success | 1539 | 4608 | 2591 | | 506 | |  | | 9244 | | 9244 |
|  | Percent within grade | 82.4% | 90.5% | 86.6% | | 73.3% | |  | | 86.9% | |  |
| Bleeding | #Valid Cases | 1913 | 5213 | 3074 | 704 | |  | | 10904 | | 10904 | |
|  | #Cases Event | 51 | 245 | 88 | 23 | |  | | 407 | | 407 | |
|  | Percent within grade | 2.7% | 4.7% | 2.9% | 3.3% | |  | | 3.7% | |  | |
| Bleeding clinically relevant | #Valid Cases | 1913 | 5213 | 3074 | 704 | |  | | 10904 | | 10904 | |
|  | #Cases Event | 5 | 17 | 11 | 9 | |  | | 42 | | 42 | |
|  | Percent within grade | 0.3% | 0.3% | 0.4% | 1.3% | |  | | 0.4% | |  | |
| Perforation | #Valid Cases | 1913 | 5213 | 3070 | 704 | | 4 | | 10900 | | 10904 | |
|  | #Cases Event | 8 | 31 | 23 | 4 | |  | | 66 | | 66 | |
|  | Percent within grade | 0.4% | 0.6% | 0.7% | 0.6% | |  | | 0.6% | |  | |
| Post-ERCP pancreatitis | #Valid Cases | 1913 | 5205 | 3061 | 701 | | 24 | | 10880 | | 10904 | |
|  | #Cases Event | 49 | 204 | 98 | 28 | |  | | 379 | | 379 | |
|  | Percent within grade | 2.6% | 3.9% | 3.2% | 4.0% | |  | | 3.5% | |  | |
| Post-ERCP cholangitis | #Valid Cases | 1912 | 5205 | 3059 | 699 | | 29 | | 10875 | | 10904 | |
|  | #Cases Event | 11 | 50 | 32 | 14 | |  | | 107 | | 107 | |
|  | Percent within grade | 0.6% | 1.0% | 1.0% | 2.0% | |  | | 1.0% | |  | |
| Cardio-pulmonal adverse event | #Valid Cases | 1912 | 5207 | 3063 | 701 | | 21 | | 10883 | | 10904 | |
|  | #Cases Event | 11 | 39 | 27 | 7 | |  | | 84 | | 84 | |
|  | Percent within grade | 0.6% | 0.7% | 0.9% | 1.0% | |  | | 0.8% | |  | |
| Other adverse event | #Valid Cases | 1904 | 5180 | 3041 | 697 | | 82 | | 10822 | | 10904 | |
|  | #Cases Event | 16 | 52 | 27 | 16 | |  | | 111 | | 111 | |
|  | Percent within grade | 0.8% | 1.0% | 0.9% | 2.3% | |  | | 1.0% | |  | |
| At least one adverse event | #Valid Cases | 1904 | 5181 | 3042 | 696 | | 81 | | 10823 | | 10904 | |
|  | #Cases Event | 130 | 565 | 257 | 80 | |  | | 1032 | | 1032 | |
|  | Percent within grade | 6.8% | 10.9% | 8.4% | 11.5% | |  | | 9.5% | |  | |
