# Supplementary material for: SASE, Success and Adverse event Score in Endoscopic Retrograde Cholangiopancreatography: a Novel Grading System
Source: BMC Gastroenterol. 2023 Sep 15;23:314. doi: 10.1186/s12876-023-02942-w (PMC10504789; doi:10.1186/s12876-023-02942-w)
Supplement: Supplementary file 3 — Supplementary Material 3 [file 12876_2023_2942_MOESM3_ESM.docx]

**SASE, Success and Adverse Event Score in Endoscopic Retrograde Cholangiopancreatography. A Novel Grading System.**

*Andreas Maieron, MD, PhD, Christine Duller, ScD, PhD, Andreas Püspök, MD, PhD, Emanuel Steiner, MD, Christine Kapral, MD, PhD*

Corresponding author

Priv.-Doz. Dr. Andreas Maieron

Department of Internal Medicine 2; Gastroenterology & Hepatology

Karl Landsteiner University of Health Sciences, University Hospital of St. Pölten

Mühlweg, A - 3100 St. Pölten, Austria

E-Mail: Andreas.Maieron@stpoelten.lknoe.at

Additional file 3: Success rates in our cohort subdivided according to the ASGE grading system

|  |  | Achievement  therapeutic  target | Cannulation  desired  duct |
| --- | --- | --- | --- |
| **ERCP** | **Cases** | **Valid percent** | **Valid percent** |
| Billroth II | 179 | 63.0% | 68.3% |
| Emergency case (outside normal hours) | 525 | 90.5% | 94.8% |
| Child < 3 years | 2 | 50.0% | 100.0% |
| Previous failed/incomplete procedure | 304 | 70.7% | 80.3% |
| Level 1 |  |  |  |
| Deep cannulation of duct of interest, main papilla sampling | 1328 | 78.3% | 85.2% |
| Aspiration of bile | 148 | 71.9% | 72.5% |
| Biliary stent removal /exchange | 760 | 90.8% | 91.4% |
| Level 2 |  |  |  |
| Biliary stone extraction < 10 mm | 3627 | 93.3% | 96.4% |
| Treat biliary leaks | 208 | 88.9% | 94.0% |
| Treat extrahepatic benign / malignant strictures | 1849 | 87.5% | 91.3% |
| Place prophylactic pancreatic stents | 232 | 77.5% | 84.5% |
| Level 3 |  |  |  |
| Biliary stone extraction > 10 mm | 1408 | 89.8% | 97.7% |
| Minor papilla cannulation in p. divisum, and therapy | 79 | 74.0% | 85.3% |
| Remove of internally migrated biliary stents | 73 | 87.5% | 100.0% |
| Intraductal imaging, biopsy | 156 | 86.4% | 98.0% |
| Manage acute or recurrent pancreatitis | 428 | 84.2% | 91.5% |
| Treat pancreatic strictures | 345 | 81.4% | 91.7% |
| Remove pancreatic stones mobile and < 5 mm | 129 | 84.9% | 95.3% |
| Treat hilar tumors | 193 | 74.1% | 95.7% |
| Treat benign biliary strictures hilum and above | 390 | 84.3% | 97.1% |
| Manage suspected sphincter Oddi dysfunction | 24 | 95.8% | 100.0% |
| Level 4 |  |  |  |
| Extract internally migrated pancreatic stent | 32 | 96.8% | 96.9% |
| Intraductal image guided therapy  (eg. PDT; electrohydraulic lithotripsy) | 93 | 82.4% | 98.9% |
| Pancreatic stones impacted and/or > 5 mm | 58 | 46.4% | 92.7% |
| Intrahepatic stones | 76 | 74.7% | 95.9% |
| Ampullectomy | 51 | 85.4% | 84.0% |
| ERCP after Whipple or Roux - en - Y | 125 | 51.2% | 54.6% |
| All cases (**NOT** sum - multiple answers possible) | 10904 | 86.9% | 92.5% |
| Minima and Maxima highlighted; Abbreviations: ERCP, endoscopic retrograde cholangiopancreatography; PDT, photodynamic therapy. | | |  |
