# Supplementary material for: SASE, Success and Adverse event Score in Endoscopic Retrograde Cholangiopancreatography: a Novel Grading System
Source: BMC Gastroenterol. 2023 Sep 15;23:314. doi: 10.1186/s12876-023-02942-w (PMC10504789; doi:10.1186/s12876-023-02942-w)
Supplement: Supplementary file 4 — Supplementary Material 4 [file 12876_2023_2942_MOESM4_ESM.docx]

**SASE, Success and Adverse Event Score in Endoscopic Retrograde Cholangiopancreatography. A Novel Grading System.**

*Andreas Maieron, MD, PhD, Christine Duller, ScD, PhD, Andreas Püspök, MD, PhD, Emanuel Steiner, MD, Christine Kapral, MD, PhD*

Corresponding author

Priv.-Doz. Dr. Andreas Maieron

Department of Internal Medicine 2; Gastroenterology & Hepatology

Karl Landsteiner University of Health Sciences, University Hospital of St. Pölten

Mühlweg, A - 3100 St. Pölten, Austria

E-Mail: Andreas.Maieron@stpoelten.lknoe.at

Additional file 4: p-values for Spearman correlation in model and validation cohort

| **SASE Success** | | **Model** Cohort | | | | | **Validation** Cohort | | | | |
| --- | --- | --- | --- | --- | --- | --- | --- | --- | --- | --- | --- |
|  |  | 1 | 2 | 3 | Sum | p-value | 1 | 2 | 3 | Sum | p-value |
|  |  | high |  | low |  |  | high |  | low |  |  |
| Success Cannulation | Valid Cases | 8534 | 1861 | 225 | 10620 | < .001 | 4424 | 787 | 106 | 5317 | < .001 |
|  | Cases Success | 8100 | 1584 | 142 | 9826 |  | 4182 | 702 | 75 | 4959 |  |
|  | Percent within grade | 94.9% | 85.1% | 63.1% | 92.5% |  | 94.5% | 89.2% | 70.8% | 93.3% |  |
| Success Therapeutic Target | Valid Cases | 8517 | 1882 | 226 | 10625 | < .001 | 4413 | 780 | 105 | 5298 | < .001 |
|  | Cases Success | 7694 | 1412 | 128 | 9234 |  | 3949 | 590 | 59 | 4598 |  |
|  | Success within grade | 90.3% | 75.0% | 56.6% | 86.9% |  | 89.5% | 75.6% | 56.2% | 86.8% |  |
|  |  |  |  |  |  |  |  |  |  |  |  |
| **SASE Adverse Event** | | **Model** Cohort | | | | | **Validation** Cohort | | | | |
|  |  | 1 | 2 | 3 | Sum | p-value | 1 | 2 | 3 | Sum | p-value |
|  |  | low |  | high |  |  | low |  | high |  |  |
| Bleeding | Valid Cases | 760 | 4574 | 5568 | 10902 | < .001 | 420 | 2177 | 2824 | 5421 | < .001 |
|  | Cases Event | 9 | 135 | 263 | 407 |  | 8 | 92 | 160 | 260 |  |
|  | Percent within grade | 1.2% | 3.0% | 4.7% | 3.7% |  | 1.9% | 4.2% | 5.7% | 4.8% |  |
| Post-ERCP Pancreatitis | Valid Cases | 760 | 4561 | 5557 | 10878 | < .001 | 420 | 2177 | 2824 | 5421 | .040 |
|  | Cases Event | 8 | 139 | 231 | 378 |  | 4 | 108 | 142 | 254 |  |
|  | Percent within grade | 1.1% | 3.0% | 4.2% | 3.5% |  | 1.0% | 5.0% | 5.0% | 4.7% |  |
| At least one complication | Valid Cases | 758 | 4536 | 5527 | 10821 | < .001 | 419 | 2170 | 2814 | 5403 | < .001 |
|  | Cases Event | 35 | 377 | 619 | 1031 |  | 19 | 237 | 351 | 607 |  |
|  | Percent within grade | 4.6% | 8.3% | 11.2% | 9.5% |  | 4.5% | 10.9% | 12.5% | 11.2% |  |

Abbreviations: ERCP, endoscopic retrograde cholangiopancreatography; SASE, Success and Adverse Event Score in Endoscopic Retrograde Cholangiopancreatography.
